# Measuring Recovery and Understanding Long-Term Deficits in Balance, Ankle Mobility and Hip Strength in People after an Open Reduction and Internal Fixation of Bimalleolar Fracture and Their Impact on Functionality: A 12-Month Longitudinal Study

**DOI:** 10.3390/jcm11092539

**Published:** 2022-04-30

**Authors:** Diana Salas-Gómez, Mario Fernández-Gorgojo, Pascual Sánchez-Juan, María Isabel Pérez-Núñez, Esther Laguna-Bercero, Amaya Prat-Luri, David Barbado

**Affiliations:** 1 Escuelas Universitarias Gimbernat (EUG), Physiotherapy School Cantabria, Movement Analysis Laboratory, University of Cantabria, 39300 Torrelavega, Spain; diana.salas@eug.es (D.S.-G.); psanchezjuan@fundacioncien.es (P.S.-J.); isabel.perez@unican.es (M.I.P.-N.); mesther.laguna@scsalud.es (E.L.-B.); 2 Alzheimer’s Centre Reina Sofia-CIEN Foundation, 28031 Madrid, Spain; 3 Neurodegenerative Disease Network Biomedical Research Center (CIBERNED), 28029 Madrid, Spain; 4 Traumatology Service and Orthopedic Surgery, University Hospital “Marqués de Valdecilla” (UHMV), 39008 Santander, Spain; 5 Sports Research Centre, Department of Sport Science, Miguel Hernández University of Elche, 03202 Elche, Spain; aprat@umh.es (A.P.-L.); dbarbado@umh.es (D.B.); 6 Alicante Institute for Health and Biomedical Research (ISABIAL), 03550 Alicante, Spain

**Keywords:** balance, ankle dorsiflexion, hip strength, functionality, clinical rating, PROMs, posturography, Y-Balance test, longitudinal study

## Abstract

To analyze how balance and other physical capacities evolved after surgery in patients with a bimalleolar fracture and how these capacities and clinical variables (immobilization or unloading time) contribute to restoring patients’ functionality, 22 patients and 10 healthy people (HC) were assessed for static and dynamic balance (Y-Balance test, YBT), dorsiflexion ankle mobility (ADF_ROM_) and hip strength at 6 and 12 months after surgery. Patients’ functional status was assessed through the Olerud Molander Ankle Score (OMAS) and the American Orthopaedic Foot and Ankle Society (AOFAS) score. Twenty-one patients with ankle fractures who completed the study showed a worse static and dynamic balance at 6 months. The YBT in the anterior direction (YBT_A_) revealed balance deficits in the operated limb at 12 months compared to the non-operated limb (−5.6%) and the HC (−6.7%). They also showed a decreased ADF_ROM_ compared to the non-operated limb (−7.4°) and the HC (−11°). In addition, medium-term (6 months) deficits in abductor strength hip but no hip strength deficits were found at 12 months after surgery. Relative weight analyses showed that ADF_ROM_ and hip strength explained 35–63% of the YBT_A_ variance and AOFAS/OMAS scores. Balance, hip strength and ADF_ROM_ seem to be reliable indexes for assessing the functional status of these patients. These results could help to understand the relationship between these physical capacities and the patients’ perceived functional status.

## 1. Introduction

Ankle fractures are one of the most commonly occurring forms of trauma managed by orthopedics teams, with an incidence ranging from 71 to 187 per 100,000 people/year [1]. Ankle fractures also amount to 10% of all types of fractures, and they rank second with regard to the frequency of occurrence [2]. When the fracture is unstable, surgery is required to restore the joint congruence [3]. The surgery employed is usually an open reduction and internal fixation (ORIF), which involves a long-term recovery and therefore entails a high socio-economic impact [1,4]. After surgery, several physical capacities are impaired, reducing functional independence in performing daily life activities, which has a profound negative impact on quality of life [4,5,6,7]. Regarding recovery time, some studies report that patients still present symptoms years after surgery [8,9]. However, other studies report that people with ankle fractures (PwAF) present little pain and restriction in activities one year after surgery [10,11]. Despite their high prevalence, there is limited evidence on the long-term effects of ankle fractures after surgery [11]. In this sense, clinical and research works conducted on PwAF have used patient-reported outcome measures (PROMs) such as the Olerud Molander Ankle Score (OMAS) [12] or mixed instruments such as the American Orthopaedic Foot and Ankle Society Ankle–Hindfoot score (AOFAS_AH_) [13] in addition to radiological findings [14,15] to assess treatment success and patient follow-up [4,16,17,18]. Both questionnaires analyze the domains related to patients’ self-perceived pain and functionality in daily life activities. In addition, the AOFAS_AH_ is an important clinical rating commonly used by clinicians to assess outcomes after ankle fracture surgery. This rating system is a standardized evaluation of the clinical status of the ankle–hindfoot. It combines subjective scores of pain and function provided by the patient with objective scores based on the surgeon’s physical examination of the patient to assess sagittal motion, hindfoot motion, ankle–hindfoot stability and alignment of the ankle–hindfoot [17,18,19,20,21,22]. Although these instruments have proven their usefulness, currently, there are no well-established methods to objectively assess the progress of a patient’s rehabilitation, so a more quantitative approach that provides objective and reproducible information is needed. Combining the information obtained from objective tools and questionnaires would provide a broader and more complete picture of the rehabilitation process of PwAF. In this sense, we believe that one of the key points in determining an accurate prognosis is to carry out reliable and quantifiable monitoring of the rehabilitation process [23]. Health care professionals must use prognostic data to comprehend the recovery course and facilitate decision-making about whether, how and when to use and modify rehabilitation interventions.

Impaired balance is one of the most important factors that reduce functional capacity in PwAF [4,8,24]. Maintaining an adequate balance is essential for developing various activities such as standing, climbing stairs or walking safely [16,25]. Balance is a complex multidimensional ability [26] that is highly dependent on the conditions in which it is evaluated. Several studies in healthy people have reported a low correlation between static and dynamic balance tasks [27,28]. These results, together with those results found in PwAF showing balance impairments in different tasks [8,24], reinforce the need to perform a battery of tests, including dynamic and static tasks, to detect any long-term balance deficits. Additionally, balance deficits in PwAF have been linked with some factors directly or indirectly caused by ankle fractures, such as decreased ankle dorsiflexion range of movement (ADF_ROM_) or less hip strength. These factors contribute to altered lower limb kinematics, especially in tasks involving single-leg stance, during dynamic actions in the sagittal plane such as single-leg reaching tasks or during gait [29,30]. Thus, balance deficits associated with these factors can alter the gait cycle [9,16,31,32,33]. The alteration of the gait cycle is highly relevant in rehabilitation since it may expose the patient to modification of the load distribution on different joints, pain and increased risk of fall [16].

Based on the above-presented rationale, balance deficits, reduced ADF_ROM_ and hip muscle weakness are well recognized as crucial targets for any rehabilitation program after an ankle fracture. However, to date, it is not known to what extent the deterioration of these abilities persists over time after surgery and to what extent they are important factors for functional capacity recovery. Therefore, the first aim of the present study was to analyze the evolution of static and dynamic balance, ADF_ROM_ and hip strength in PwAF at 6 and 12 months after surgery. As a second aim of this study, we assessed to what extent ADF_ROM_; hip strength; and clinical variables such as immobilization time, unloading period and time of rehabilitation were related to patients’ balance restoration at 6 and 12 months after surgery. Similarly, the relationship between physical capacities, clinical variables and the OMAS and AOFAS_AH_ results was explored to understand how balance, ADF_ROM_ and hip strength restoration influence patients’ perception of disability. Finally, due to the impact ADF_ROM_ and hip strength have on balance and other functional activities, the extent to which the restoration of these variables was affected by the clinical variables was also explored.

## 2. Materials and Methods

### 2.1. Participants

Twenty-two PwAF were recruited from the Traumatology Unit of the Marqués de Valdecilla University Hospital (HMV). All participants underwent ankle surgery (ORIF) conducted by the same surgeons (2 surgeons) followed by a similar post-surgical intervention consisting of immobilization and unloading periods followed by a rehabilitation program. Patients were selected through their medical records, and those who met the inclusion criteria were invited by their surgeons to participate after surgery. Those individuals that (1) had prior lower-limb surgery; (2) had bilateral ankle affection; (3) had a neurologic or rheumatologic disease; (4) had an open fracture, pathological fracture or tibial pilon fracture; (5) had not completed the follow-up period; or were (6) younger or older than 18 or 55 years, respectively, were excluded from the study. Likewise, 10 HC of similar age and gender took part in this study. The exclusion criteria for being part of the control group were as follows: (1) patients having received previous surgery on the lower extremities, (2) patients who suffered neurological or rheumatic pathology, (3) patients under 18 years of age and over 55 years of age and (4) patients with any type of fracture located in the lower limb. An a priori sample size calculation was performed to estimate the minimum number of subjects that were needed for the study. The minimum number of subjects required to detect differences between groups was calculated using mixed ANOVA (one intergroup factor with two levels: ankle fracture–healthy control; one intragroup factor with two levels: limb: operated (dominant)–non-operated (non-dominant)). Sample calculation was performed using the G*Power 3.1.9.2 software package, and it was based on the effect size estimate from a previous study [34]. This study found differences in dynamic balance in subjects with chronic ankle instability and healthy subjects (d = 0.77). Based on these results, the authors expected to find differences in dynamic balance with a large effect size between the operated limb of patients with ankle fractures and healthy subjects. Conversely, we did not expect to find significant differences between the non-operated limb of patients with ankle fractures and healthy controls. Based on this criterion, 20 participants (10 per group) were necessary to detect a significant interaction effect between groups and limbs (effect size f = 0.35; power = 80%; α = 0.05, correlation between repeated measures, r = 0.5). Eleven healthy people were recruited and one person was eliminated due to errors in the assessment. In addition, a sample calculation was also performed to determine the minimum number of subjects with an ankle fracture. Based on the effect size estimation (g = 0.7) of previous studies [19,35], a sample size of 19 participants was found to be necessary to detect between-limb differences (power = 80%; α = 0.05). Twenty-two patients were recruited to allow up to a 10% dropout rate. Informed consent was completed by all participants prior to the start of the study, which was approved by the Cantabria Clinical Research Ethics Committee of the Marqués de Valdecilla University Hospital (reference: 2017.072). The procedures were in line with the Declaration of Helsinki.

### 2.2. Experimental Procedure

**Figure 1:**
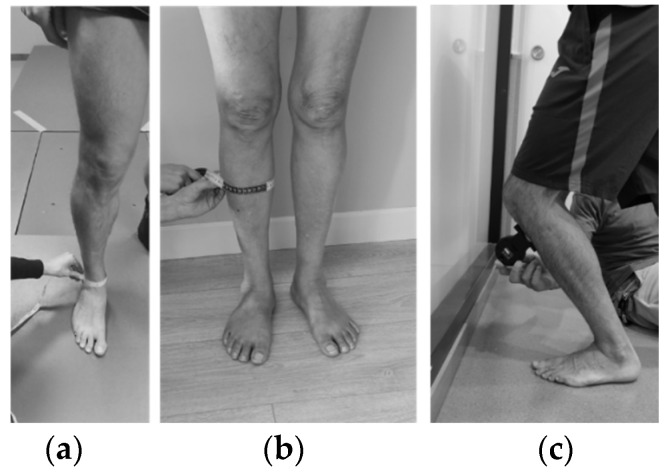
(**a**) Measurement of bimalleolar circumference; (**b**) measurement of calf circumference; (**c**) position of ankle dorsiflexion range of movement measurement (weight-bearing lunge (WBL) method with the knee bent).

**Figure 2:**
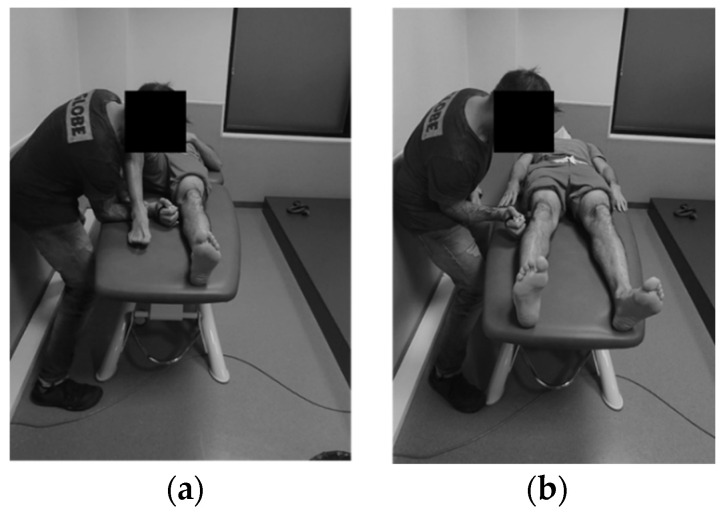
(**a**) Adductor hip strength assessment; (**b**) abductor hip strength assessment.

This was a longitudinal prospective study that was carried out in a movement analysis laboratory. Two assessment sessions (2 h duration each) were carried out 6 and 12 months after the bimalleolar ankle surgery of each patient. Both sessions had the following structure, and all measurements and tests were performed bilaterally on the lower extremities: (1) collection of descriptive and anthropometric data (i.e., body mass, height, limb length and bimalleolar and calf circumferences [36] (Figure 1); (2) completion of the AOFAS_AH_ and the OMAS questionnaires; and (3) assessment of ADF_ROM_, hip strength and balance. In addition, clinical data were collected from the patients’ clinical histories, which were provided by their surgeons (i.e., type of fracture according to the AO Foundation and Orthopedic Trauma Association (AO-OTA) [37], the immobilization time, the unloading period, AOFAS_AH_ alignment, the rehabilitation time and information about rehabilitation sessions). Other variables (type of ORIF, injury mechanism and post-surgical complications) were also registered.

#### 2.2.1. Functional Status Questionnaires

The AOFAS_AH_ and the OMAS questionnaires were employed to assess the functional status after ankle surgery. The AOFAS_AH_ questionnaire score reaches a maximum of 100 points, with higher scores representing higher functionality. It is made up of three domains referring to pain (up to 40 points), function (up to 50 points) and alignment (up to 10 points). The last domain is assessed by the orthopedic surgeon. The study conducted by de Boer et al. on patients with malleolar fractures reported a high test–retest reliability for the total AOFAS and its AOFAS domains with intraclass correlation index (ICC) values of 0.85 ≤ ICC ≤ 0.93, standard error of measurement (SEM) values of 0.9 ≤ SEM ≤ 4.3 points and minimum detectable change (MDC) values of 2.6 ≤ MDC ≤ 12.0 points [22]. In addition, normative values above 90 points have been reported for people up to the age of 80 years [21]. The score is categorized as follows: excellent, from 90 to 100 points; good, from 80 to 89 points; fair, from 60 to 79 points; and poor, less than 60 points [38]. In the same way, the OMAS questionnaire score reaches up to 100 points, with a higher rating associated with a better functional status. The OMAS has shown an ICC value of 0.98, with an SEM of 3.3 points and MDC of 9.1 points [39]. OMAS scores are categorized as follows: excellent, from 91 to 100 points; good, from 61 to 90 points; fair, from 31 to 60 points; poor, less than 30 points [39].

#### 2.2.2. Ankle Dorsiflexion Range of Movement

A digital inclinometer, which was placed on the participant’s distal part of the tibial tuberosity to increase the test reliability, was employed to assess ADF_ROM_ (Acumar, Lafayette Instrument, Lafayette, IN, USA). Specifically, ankle ROM was measured using the weight-bearing lunge (WBL) method with the knee bent according to the procedure previously described [40,41]. This method has shown very high reliability (ICC > 0.9), with an SEM from 1.3° to 1.4° and MDC from 3.7° to 3.8° [41,42]. Three attempts were allowed for each limb. The participants stood barefoot, facing the wall, with the foot that was going to be assessed placed 30 cm from the wall, keeping their arms in contact with the wall and their knee aligned with the second toe. From that position, participants bent their knees toward the wall until they reached their maximum ADF_ROM_ (Figure 1).

#### 2.2.3. Hip Strength

The maximal force exerted during the hip abduction (H_ABD_) and adduction (H_ADD_) isometric contractions was registered with a hand-held dynamometer (HHD) (microFET@2, Hoggan Scientific L.L.C, Salt Lake City, UT, USA) [43]. The participants lay in a supine position on a stretcher with their knees extended and their arms parallel to their bodies. Participants carried out a warm-up consisting of two progressive trials before testing. Afterward, they performed three trials with a one-minute rest between each. Participants were asked to reach their maximal force progressively in a 5 s window. They were verbally encouraged during each trial. This method has shown high reliability (ICC > 0.9), with an SEM from 1.0% to 1.8% and MDC from 2.6% to 5.4% (SEM and MDC are in force units normalized by the body mass) [44,45]. HHD has previously been shown to be valid and comparable with the criterion standard in strength testing and isokinetic dynamometry, without sacrificing ease of use, portability or affordability (Figure 2) [43].

#### 2.2.4. Balance

**Figure 3:**
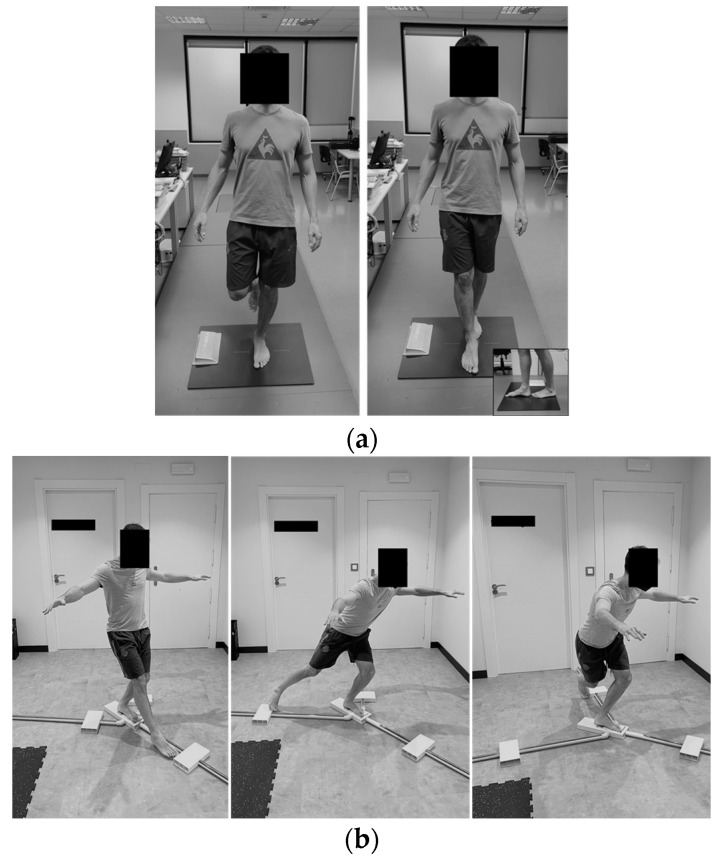
(**a**) Posturographic assessment of static balance (from left to right): unipodal support position, tandem position; (**b**) assessment of the dynamic balance (left limb support, from left to right): Y-Balance test anterior direction, Y-Balance test posteromedial, Y-Balance test posterolateral.

Static and dynamic balance were assessed through static tasks on a pressure platform (P-Walk, BTS Bioengineering, Milano, Italy) [46] and the Y-Balance test (YBT) (Y-Balance Test Kit, Move2Perform, Evansville, IN, USA) [47], respectively. The pressure platform provides quantitative information by analyzing the center of pressure (CoP) oscillations during each test. Quantitative analysis of the CoP parameters can be used to identify balance disorders. The balance study based on CoP parameters has shown high reliability (>0.8) [48]. The sampling frequency was 100 Hz. The pressure platform was remotely controlled with the “G-studio” software (BTS Bioengineering, Milano, Italy). Participants carried out four balance tasks in the following order: (1) single-leg stance with the non-operated limb and the eyes open (SL-NOL_EO_), (2) single-leg stance with the operated limb and the eyes open (SL-OL_EO_), (3) tandem position with the non-operated limb behind the operated limb and the eyes open (TD-NOL_EO_) and (4) tandem position with the operated limb behind the non-operated limb and the eyes open (TD-OL_EO_). Trials were alternated between the non-operated and the operated limb to avoid fatigue. Before testing, participants had a brief familiarization period consisting of trying out each of the positions and conditions. Afterward, they performed two testing trials that lasted 30 s with a 30 s rest between trials. During the assessment of these tasks, a researcher was placed in front of the participants to avoid any possible fall. Participants were allowed to perform movements with their arms (Figure 3).

Participants’ dynamic balance was assessed with the YBT. The YBT has been shown to be an instrument with high-to-excellent reliability for assessing dynamic balance in various populations with ankle injuries [44,47,49]. The tool kit used to carry out this task consists of a central plastic plate on which the support foot must be placed, with three tubes attached in anterior, posteromedial and posterolateral directions. Each tube has a plastic box that can be moved along it. Participants performed the test barefoot in anterior (YBT_A_), posteromedial (YBT_PM_) and posterolateral (YBT_PL_) directions with both limbs. While maintaining a single-leg stance, the participants were instructed to “reach the farthest distance they could using their free foot without lifting the heel of the supporting limb and then return to the starting point without losing their balance”. A brief familiarization period of two trials in all directions was performed. Then, the testing trials were performed with 20 s of rest between trials. The participants were allowed to make as many attempts as they needed until they performed two valid trials. Trials were alternated between the non-operated and the operated limb in order to avoid fatigue. A trial was considered unsuccessful if (i) the non-tested limb touched the floor; (ii) the participant placed his or her foot on the top of the box to reach further; or (iii) the participant flicked or kicked the box, losing contact with it during the thrust phase to reach further (Figure 3).

#### 2.2.5. Data Reduction

For the ADF_ROM_ test, the two most similar trials were averaged [40]. Regarding H_ABD_ and H_ADD_ strength, the highest peak of force obtained in any trial was selected for the subsequent statistical analyses. The H_ABD_ and H_ADD_ peak of forces were normalized to each participant’s body mass (kg × 100/body mass). The total CoP length path (DIS_COP_) (mm), the average speed (MV_COP_) (mm/s) and the length/surface (LFS) were calculated and averaged from both trials of each static balance task performed on the pressure platform. The YBT was registered in centimeters and normalized to each participant’s limb length (distance reached × 100/limb length). Limb length was measured from the anterosuperior iliac spine to the medial malleolus. The best result in each direction was used for the subsequent analyses. Furthermore, a composite score (YBT_CS_) was calculated as the average of the maximum normalized distance reached in the three directions [47].

### 2.3. Statistical Analysis

Descriptive data were calculated (mean and standard deviation) for all the outcomes assessed. Normal data distribution was checked with the Shapiro–Wilk statistic. The comparison between groups was analyzed with respect to socio-demographic and anthropometric variables. Two-way repeated-measures analyses of variance (ANOVAs) were performed to analyze the differences between the operated and the non-operated limb at 6 and 12 months after surgery. Two-way mixed ANOVAs were performed to analyze the differences between the operated and the non-operated limb compared to the control group (dominant and non-dominant limbs). In addition, the asymmetries (difference between limbs) between groups at 6 and 12 months after surgery were assessed via ANOVAs. For this scope, the operated limb was compared with the dominant limb of the HC [50]. The magnitude of the differences was quantified through Hedges’ g (g) as effect size index according to the following interpretation: trivial (g < 0.2), small (0.2 ≤ g < 0.5), moderate (0.5 ≤ g < 0.8) and large (g ≥ 0.8) [51]. For Hedges’ g, negative scores indicate a lower performance of the operated limb of PwAF compared to the non-operated limb of PwAF or to the HC. Furthermore, Pearson correlation analyses were performed to observe which parameters were associated at 6 and 12 months after ankle surgery. Correlational analyses were performed between age, physical capacities, clinical variables, AOFAS_AH_ total score_,_ AOFAS domain function and OMAS and those balance tasks that showed significant differences between the limbs at 6 and 12 months after surgery. The SPSS statistical package (version 20.0, SPSS Inc., Chicago, IL, USA) was used for the ANOVAs as well as for the correlations. The significance level for the analyses was established at *p* < 0.05.

Finally, relative weight analyses (RWAs) [52] were performed to evaluate the relative contribution of the different outcome measures in this study in explaining the total variance (R^2^) of those balance tasks that showed significant differences in the evaluation session at 12 months after surgery, as well as the AOFAS_AH_ total score_,_ AOFAS domain function and OMAS questionnaire scores. The age, the clinical variables and those parameters recorded at 6 months after surgery showing a significant correlation with those above-mentioned dependent variables at 12 months after surgery were introduced as predictor variables in each RWA model. In addition, an RWA was performed to evaluate the impact and relative importance of clinical variables on the restoration of ADF_ROM_ and hip strength. A regressive elimination procedure was used to remove all parameters that did not influence the dependent variables (*p* > 0.05). All potential factors met the assumptions of normality and homoscedasticity. The RWA web [53] was used for this analysis.

## 3. Results

Twenty-one participants (10 females and 11 males) that had undergone surgery after a bimalleolar ankle fracture finished the study (Table 1). The mechanisms of injury were 14% ankle sprains, 62% falls, 9.5% traffic accidents, 9.5% falls with ankle sprains and 4.8% practicing sport. Fracture types following the OA-OTA classification are shown in Table 1. The immobilization period after surgery ranged from 1 to 6 weeks, and the period of unloading ranged from 2 to 8 weeks. In five cases, the syndesmosis was closed with suprasyndesmotic screws through the plate, and only in one patient it was removed after 4 months to improve ankle dorsiflexion. The time of rehabilitation was, on average, 3.1 months (Table 1). During the first 6 weeks, the rehabilitation program consisted of passive stretching, kinesitherapy and dorsiflexion strengthening exercises. Once the orthopedic surgeon team authorized the progressive loading phase, participants performed a static balance, proprioceptive and walking training program. The duration was established by the hospital rehabilitation service based on individual symptoms and therefore varied in each case. There were no complications. General data comparison between the two groups shows no difference, as shown in Table 1.

### 3.1. Functional Status

**Figure 4:**
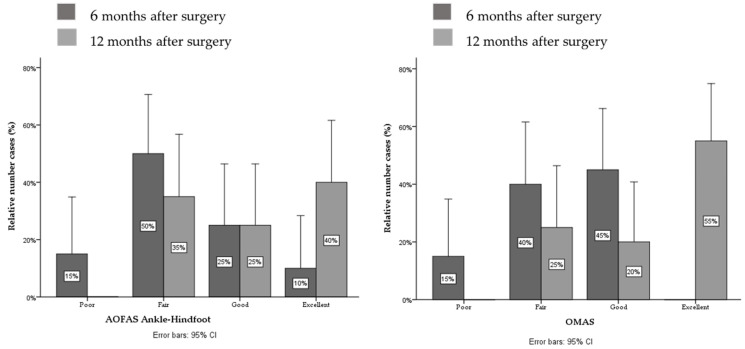
From **left** to **right**: AOFAS (American Orthopaedic Foot and Ankle Society) Ankle–Hindfoot and OMAS scores in categories. The bars represent the number of cases (%) that had poor, fair, good and excellent results at 6 and 12 months after surgery.

Participants showed significant improvement in scores in both AOFAS_AH_ (∆ = +10.8 points, g = 0.8) and OMAS (∆ = +22.6 points, g = 0.9) questionnaires comparing 6 months to 12 months after surgery assessments (Table 2). Based on the AOFAS_AH_ at 6 months after surgery, 50% of the patients showed fair results and only 10% showed excellent results. According to the OMAS at 6 months after surgery, 40% of the patients showed fair results and 45% showed good results. At 12 months after surgery, only 40% of the patients showed excellent results in the AOFAS_AH_ and 55% in the OMAS (Figure 4).

### 3.2. Evolution of Static and Dynamic Balance

**Figure 5:**
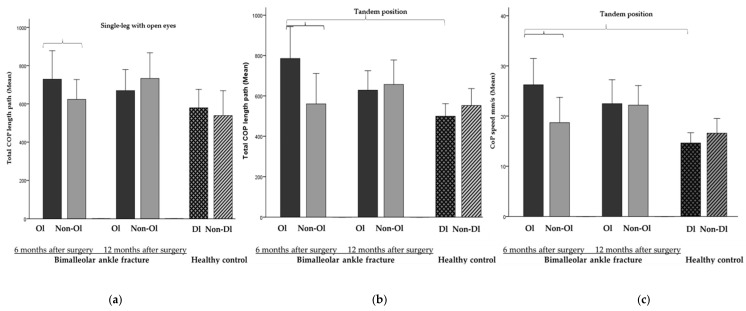
Static balance results (from left to right): **Ol:** Operated limb; **Non-Ol:** Non-operated limb; **Dl:** dominant liimb; **Non-Dl:** Non-dominant limb. (**a**) Single-stance and (**b**) tandem position: >total centre of pressure (CoP) length path and (**c**) tandem position: >CoP speed in operated limb compared to non-operated limb at 6 months after surgery in people with bimalleolar ankle fracture (−0.3 ≤ g ≤ −0.7, respectively); (**b**,**c**) tandem position: (**b**) total CoP length path and (**c**) CoP speed in operated limb of people with bimalleolar ankle fracture compared to dominant limb of healthy control at 6 months after surgery (g = −0.8 and g = −0.8, respectively). Error bars: 95%.

All the patients were able to perform all the different static tasks. At 6 months after surgery, a worse performance was detected in the operated limb compared to the non-operated limb in the single-leg stance with open eyes and the tandem tasks (SL-OL_EO_ > DIS_COP_, g = −0.3; TD-OL_EO_ > DIS_COP_, g = −0.6 and >MV_COP_, g = −0.7) (Figure 5 and Table 3). However, no significant differences between limbs were observed at 12 months after surgery.

Compared to the HC, PwAF showed worse static balance performance in their operated limb at 6 months after surgery (TD-OL_EO_ > DIS_COP_, g = −0.8 and >V_COP_, g = −0.8) but no differences were observed at 12 months (Figure 5 and Table 3).

Regarding the dynamic balance at 6 months after surgery, all patients were able to perform the YBT_A_, 20 of them were able to perform the YBT_PM_ and 19 were able to perform the YBT_PL_. At 12 months after surgery, two subjects were still not able to perform the YBT_PL_ as they failed to meet some of the criteria described above in the methodology to count a test as valid. At 6 months after surgery, PwAF showed lower results for the operated limb in the YBT_A_ (−9.4%, g = −0.7) and the YBT_CS_ (−4.7%, g = −0.5) compared to the non-operated limb. Patients showed a significant increase in the distance reached by the operated limb in both tasks (YBT_A_: ∆ = +4.9%, g = 0.3; YBT_CS_: ∆ = +3.5%, g = 0.3) at 12 months after surgery; however, the patients still presented lower significant balance performance in their operated limb compared to their non-operated limb (YBT_A_: −5.6%, g = −0.5; YBT_PM_: −3.3%, g = −0.3; YBT_CS_: −3.3%, g = −0.3).

**Figure 6:**
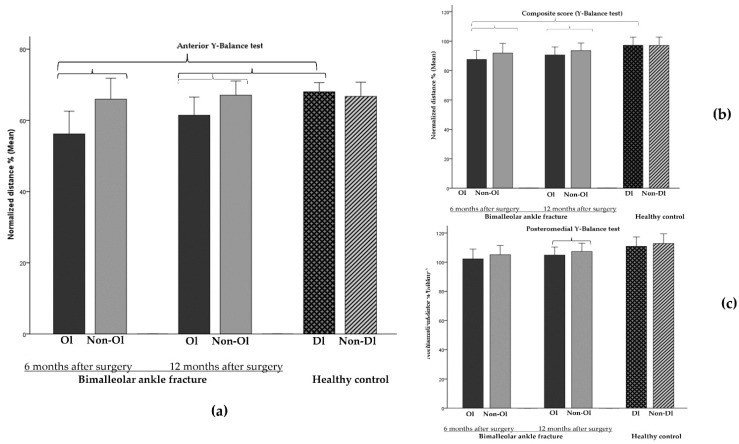
Dynamic balance results (Y-Balance test): **Ol:** operated limb; **Non-Ol:** Non-operated limb; **Dl:** dominant limb; **Non-Dl:** Non-dominant limb. (**a**) distance reached in Y-Balance test anterior direction in operated limb compared to non-operated limb at 6 and 12 months after surgery in people with bimalleolar ankle fracture and distance reached in operated limb of people with bimalleolar ankle fracture compared to dominant limb of healthy control at 6 and 12 months after surgery (−0.5 ≤ g ≤ −0.9); (**b**) composite score in operated limb compared to non-operated limb at 6 and 12 months after surgery in people with bimalleolar ankle fracture and distance reached in operated limb of people with bimalleolar ankle fracture compared to dominant limb of healthy control at 6 months after surgery (−0.3 ≤ g ≤ −0.9); (**c**) distance reached in Y-Balance test posteromedial direction in operated limb compared to non-operated limb at 12 months after surgery in people with bimalleolar ankle fracture (g = −0.3). Error bars: 95% CI.

Compared to the HC, PwAF showed significant lower scores in the YBT_A_ (−11.6%, g = −1.0) and the YBT_CS_ (−10.0%, g = −0.9) at 6 months after surgery for the operated limb but not for the non-operated limb (interaction effect, F = 15.895, *p* = <0.0001 and F = 7.098, *p* = <0.001, respectively). PwAF still presented poorer balance at 12 months after surgery in the YBT_A_ (−6.7%, g = −0.7) compared to the HC (interaction effect, F = 7.310, *p* = 0.01) (Figure 6 and Table 4).

### 3.3. Evolution of Ankle ROM and Hip Strength and Circumferences

**Figure 7:**
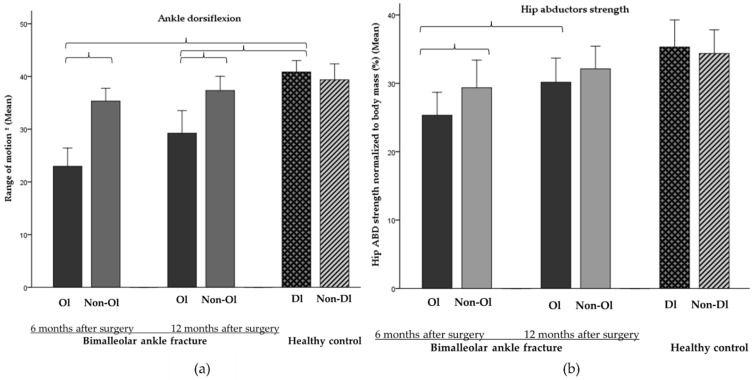
**Ol:** operated limb; **Non-Ol:** Non-operated limb; **Dl:** dominant limb; **Non-Dl:** Non-dominant limb. (**a**) Ankle dorsal flexion ROM. Range of dorsal flexion of the operated limb at 6 and 12 months after surgery in people with bimalleolar ankle fracture compared with non-operated limb and with the dominant limb of the healthy control (−0.8 ≤ g ≤ −2.5). (**b**) Hip abductor strength of the operated limb at 6 months after surgery in people with bimalleolar ankle fracture compared with non-operated limb and with the dominant limb of the healthy control (−0.5 ≤ g ≤ −1.2). Error bars: 95% CI.

The operated limb of PwAF showed lower ADF_ROM_ than the non-operated limb at 6 months after surgery (−12.4°, g = −1.7). The operated limb showed an increase in ADF_ROM_ in follow-up (∆= +6.8°, g = 0.8), but the differences between limbs were still significant at 12 months after surgery (−7.4°, g = −0.8). Compared to the HC, the operated limb of PwAF showed lower ADF_ROM_ at both evaluations (−18.7°, g = −2.5 and −11°, g = −1.3, respectively), but this difference was not observed for the non-operated limb (interaction effect, F = 63.104, *p* ≤ 0.0001 and F = 19.931, *p* ≤ 0.001, respectively). Regarding the hip muscle strength, PwAF showed lower H_ABD_ strength in the operated limb compared to the non-operated limb at 6 months (−4.1%, g = −0.5) but not at 12 months after surgery. Compared to the HC, PwAF had lower H_ABD_ strength (−9.7%, g = −1.2) in the operated limb but not in the non-operated limb (interaction effect, F = 6.291, *p* = 0.02) at 6 months but not at 12 months after surgery (Supplementary Table S1). The results of the dorsal ankle flexion and hip strength are shown in Figure 7 and Table S1. The calf and bimalleolar circumferences results are shown in Table S1.

### 3.4. Correlation Analysis between Balance, Physical Capacities, Clinical Variables and Questionnaires

A lower distance achieved in the YBT_A_ at 6 and 12 months after surgery was significantly associated with a lower ADF_ROM_ (0.535 ≤ r ≤ 0.685), lower H_ABD_ and H_ADD_ strength (0.613 ≤ r ≤ 0.773) and longer rehabilitation time (−0.448 ≤ r ≤ −0.516). Similar results were found for the YBT_CS_ (Supplementary Table S2). Concerning static balance, only at 6 months after surgery, TD-OL_EO_ revealed an association with ADF_ROM_ (r = −0.432), time of immobilization (r = 0.476) and period of unloading (r = 0.446), which indicated that the DIS_COP_ covered was larger with a lower ADF_ROM_, longer time of immobilization and longer period of unloading (Table S2).

At 6 and 12 months after surgery, low AOFAS_AH_ scores mainly in the function domain were significantly associated with low YBT_A_ (0.454 ≤ r ≤ 0.674), reduced ADF_ROM_ (0.479 ≤ r ≤ 0.751) and lower hip strength (0.472 ≤ r ≤ 0.716). At 6 and 12 months after surgery, low OMAS scores were associated with similar parameters (Table S2). Lower values in total AOFAS_AH_ scores and its domains were also associated with a longer rehabilitation time at 6 months after surgery (−0.640 ≤ r ≤ −0.815).

In addition, at 6 and 12 months after surgery, lower ADF_ROM_ was associated with a longer time of immobilization (−0.438 ≤ r ≤ −0.480) and a longer period of unloading (−0.467 ≤ r ≤ −0.561); lower ADF_ROM_ was also associated a longer rehabilitation time, but only at 6 months after surgery (r = −0.598) (Table S2). Finally, we observed that age was inversely correlated with some tasks of dynamic balance, namely YBT_A_, YBT_PM_ and YBT_CS_ (−0.500 ≤ r ≤ −0.717), and with lower hip strength (−0.454 ≤ r ≤ −0.499) at 6 and 12 months; age was also inversely correlated with lower AOFAS_AH_ scores, but only at 6 months after surgery (r = −0.467) (Table S2).

### 3.5. Relative Weight Analysis

RWAs were carried out on the YBT_A_, YBT_PM_, YBT_CS,_ AOFAS_AH_, OMAS scores and ADF_ROM_ observed 12 months after surgery. Age was introduced in the RWAs after observing simple correlations with some variables. For the YBT_A_ model (63% total variance explained), the variables with a significant weight were ADF_ROM_ and hip strength, but none of the clinical variables showed a significant weight. The YBT_PM_ and YBT_CS_ models included age and H_ABD_ (Table 5). Regarding ankle functionality questionnaires, only hip strength showed a significant weight in the AOFAS_AH_ model (44% total variance explained). In the functional domain of the AOFAS_AH_ score, the total variance explained was 47%. The model included the ADF_ROM_, the H_ABD_ and the H_ADD_ strength (Table 5). The OMAS model (35% total variance explained) only included H_ADD_ strength and H_ABD_ strength as significant predictors. For the ADF_ROM_, the total variance explained was 55%, and only the immobilization time and the unloading period had a significant relative weight. None of the clinical variables were predictors of hip strength. None of the parameters showed a significantly higher weight than others in predicting any of the RWAs (Table 5).

## 4. Discussion

This study analyzed how balance, ankle dorsiflexion and hip strength are affected and how they progress in PwAF at 6 and 12 months after surgery. As a second main aim, the relationship that physical abilities and clinical variables have with ankle functionality questionnaires was explored to understand the impact of these factors on the functional recovery of PwAF.

### 4.1. Evolution of Balance, Ankle ROM and Hip Strength from 6 to 12 Months after Surgery

Our results showed that PwAF presented balance deficits in their operated limb compared to their non-operated limb at both 6 and 12 months after surgery; however, these deficits were mainly observed when the balance was assessed in dynamic conditions. Specifically, the most significant balance impairment was observed when participants performed the anterior reaching direction of the YBT with a bilateral deficit of 9.4% and 5.6% at 6 and 12 months after surgery, respectively. Although the evidence in PwAF is limited, our results are supported by previous findings showing the YBT to be a suitable and sensitive tool to detect balance impairments caused by ankle fractures [19,44] or less severe ankle injuries [34,49]. However, it is important to note that in PwAF, the between-limb asymmetries persisted significantly at 12 months after surgery in YBT_A_ (5.6%). This coincides with previous studies that have also detected between-limb asymmetries in subjects with ankle injuries [34,54]. In line with what was previously reported, no asymmetries were detected in the HC [55]. Therefore, these results confirm that PwAF presented long-term balance impairments and asymmetries between limbs after surgery and that challenging tests are needed to detect those balance deficits. Clinically, it is important to be able to quantify and address these asymmetries, as other authors have reported that these asymmetries can lead to a risk of injury [56,57].

Conversely, when balance was assessed through static posturographic tasks, few differences between limbs were observed, and these were found at 6 but not at 12 months after surgery. Therefore, it seems that balance differences between limbs in static tasks are normalized at 12 months after surgery, which seems to be supported by previous works that have found minimal (less than the 10% that has been reported in healthy subjects) or no long-term static balance differences between limbs in PwAF [8,58].

Finally, although the operated ADF_ROM_ improved significantly in the follow-up, the asymmetries between limbs (7.43°) persist at 12 months after surgery. This finding is in agreement with previous works that reported a long-term ADF_ROM_ limitation after surgery [19,59,60]. As for limb asymmetries in hip strength, a lower abduction strength was observed in the operated limb at 6 months, but at 12 months after surgery, the values were similar. Although our results are in a limited sample, it seems important to address between-limb deficits in hip strength and/or ADF_ROM_ in order to minimize long-term impairment, since they can lead to between-limb asymmetries that negatively impact tasks such as walking [9,16,31].

### 4.2. Correlation Analysis between Balance, Physical Capacities, Clinical Variables and Questionnaires

We observed that low H_ABD_ and H_ADD_ and reduced ADF_ROM_ were related to low YBT_A_ and YBT_CS_ scores in PwAF, which confirms the relevance that ankle flexibility and hip muscle strength in single-leg dynamic balance tasks have in different populations such as PwAF [19,44] or people with ankle instability [29,32,33,61,62]. Interestingly, we also observed that a poor balance performance during the TD-OL_EO_ was associated with a reduced ADF_ROM_ but not with lower hip strength. One hypothesis that could explain why YBT_A_ and TD-OL_EO_ have different associated parameters is related to the way that balance is restored depending on the magnitude of the postural disturbances and the support base. During the TD-OL_EO_, the ankle control is sufficient to handle the small body disturbances that occur during this task (i.e., ankle strategy), so ankle physical status is key to a proper balance performance [63]. For example, based on our results, poor ankle flexibility may hinder the transfer of the body weight from the operated limb to the non-operated limb as a strategy to maintain balance in this position [64]. However, performing reaching dynamic actions on a single leg (i.e., YBT) requires the active participation of the hip (i.e., hip strategy) to handle larger balance perturbations [63]. To confirm this hypothesis, future studies should evaluate the neuromuscular contributions of the ankle and hip complex using electromyography during different balance tasks in PwAF.

Regarding functional questionnaires, a previous work observed that ADF_ROM_ was the factor most correlated with OMAS 14 months after ankle surgery [8]. Our correlational results observed in the OMAS and AOFAS_AH_ scores seem to support these findings; nevertheless, dynamic balance and hip strength also appeared as highly correlated factors. These findings are not surprising, as other authors have reported that both the ankle and hip neuromuscular complexes are important for a person’s mobility and for performing functional skills [29,65,66]. Regarding the calf circumference measurement, we expected to find an association with the functional status of the PwAF, as this parameter is considered a good predictor of muscle mass and, therefore, muscle strength [67]; however, we did not find any significant correlation.

### 4.3. Relative Weight Analyses

We performed RWAs to explore how clinical and physical parameters obtained 6 months after surgery comprehensively predict the physical and functional status of PwAF 12 months after surgery. Our results showed that the ADF_ROM_ and hip strength at 6 months explained a remarkable variance in the AOFAS_AH_’s domain of function. However, only hip strength showed a significant variance explanation for the prediction of OMAS and AOFAS_AH_ total scores at 12 months after surgery. It was found that similar results predicted the dynamic balance performance of PwAF at 12 months after surgery, especially when the YBT test was performed in the sagittal plane. Therefore, the ADF_ROM_ and hip strength observed in the medium term seem to be predictive variables for better long-term functionality and balance. Although more studies with larger sample sizes are needed in this line of research, our results confirm the predictive character of both ankle ROM and hip muscle strength for balance, as has been shown in different populations [29,65,68]. It is important to highlight the relevance of hip strength, which is not a physical parameter directly affected by the ankle fracture and the subsequent surgery, on patients’ balance and functional status. As it has been well documented by previous works carried out in people with chronic ankle instability [61,62], our findings reinforce the prominent role of hip strength in dynamic balance when an ankle deficit exists, and therefore, hip strengthening exercises could be included in a comprehensive rehabilitation program in this population.

On the other hand, it should be noted that in the multivariate analysis (RWA), age was not shown to be a significant predictor of worse or better functional status or dynamic balance (YBT_A_), as has been previously reported [8]. Age only had a significant weight in the performance of the YBT_PM_ and YBT_CS_, which were not the most affected in the PwAF in this study. Therefore, it appears that the deficits in YBT_A_ in this population are caused by the direct or indirect sequelae of the fracture.

It is also interesting to point out that clinical variables (e.g., immobilization time, unloading period, rehabilitation time) were not shown to be significant predictors for a better outcome in the functional status or balance multivariate models. These results are supported by Hancock et al., who did not observe an association between the rehabilitation time and better outcomes in PwAF. In this sense, these authors suggest that the lack of relation of these variables may be biased by the fact that the fractures that required longer rehabilitation time were the most severe, which could also be the case in our study [69]. On the other hand, since an insufficient or unspecific rehabilitation might be a cause of long-term disability in these patients [10], targeted treatment focused on the factors associated with better or worse prognosis seems to be a key factor in optimizing rehabilitation processes. Our results show that only half of the patients show excellent results one year after surgery. Similar results have been reported in the literature [8,16,70]. Based on our results, it would be interesting to evaluate whether an increase in the specific rehabilitation of static and dynamic balance could improve medium- and long-term results and to evaluate its impact on functional activities such as walking [16,25].

An important issue that we think is relevant to emphasize is the finding that the immobilization and unloading time was inversely correlated [71] with the recovery (55% of explained variance) of ADF_ROM_ at 12 months after surgery. In this regard, current evidence seems to support that early mobilization and weight-bearing would reduce hospital stay, speed up return to work and sport practice, and promote early ROM recovery without resulting in an increase in complications [72,73,74,75].

### 4.4. Limitations

This study presents the natural limitations of a prospective study that could bias the result interpretations presented above. Firstly, as the main objective was to understand the evolution of balance and the other parameters analyzed after an ankle fracture surgically treated in an ecological context, there was no control of some variables such as the level of physical activity of the participants and work, which could have a great impact on the recovery process. Second, although the sample size could be considered adequate for a biomechanical study that includes a posturographic assessment, it was still too low to provide a normative reference for the magnitude of correlational analyses (i.e., Pearson correlations and RWAs) [76]. Another limitation was the difference in the group sizes of the sample. Although the recruitment of the control group subjects was performed based on a sample calculation, we believe that having equal-sized groups would have improved the quality of the results.

Finally, other physical capacities such as ankle strength or other muscles of the limb or trunk, which can be highly important and influence functional recovery, were not assessed. Based on the aforementioned concerns, we consider that it would be of clinical interest to carry out the study in a larger cohort of subjects and to resolve some of the current limitations. Future long-term randomized controlled trials should also be carried out to overcome the limitations and to investigate the real impact of the physical and functional parameters pointed out in this study.

### 4.5. Clinical Implication

Addressing balance problems is an essential factor for the recovery of the functionality of PwAF. According to our results, the static and dynamic balance tests may be sensitive tools to detect long-term balance deficits and asymmetries between limbs in patients with ankle fractures, but they can also detect deficits when these patients are compared to healthy individuals. These instruments of assessment can provide objective and reproducible information about the alteration of balance and its impact on functional status. Therefore, they present an important clinical value in assessing the balance status and progression of PwAF in rehabilitation and post-rehabilitation programs. The patients in this study not only show deficits in the ankle due to the ankle fracture but also present medium-term deficits in distal zones (hip). For this, due to the impact of ADF_ROM_ and hip strength on dynamic balance tasks, it is also advisable to address them through hip strength and ankle flexibility exercises. Regarding balance exercises, our results suggest that exercises such as the YBT could be implemented, as a person’s ability to perform this task depends on a successful combination of factors such as range of motion (ROM); neuromuscular control; and strength of the ankle, knee and hip muscles, which, in turn, can be potentially beneficial in gait rehabilitation or other functional activities of people with ankle fractures [49,77,78].

## 5. Conclusions

PwAF showed worse balance in their operated limb, especially when balance was assessed through a single-leg dynamic test performed in the anterior reaching direction (YBT_A_). PwAF also had a limitation in the ADF_ROM_. In addition, they presented medium-term deficits in abductor strength hip.

Likewise, proper YBT_A_ performance was associated with a high ankle dorsiflexion range of motion and the strength of the hip abductor and adductor muscles. The RWA showed that immobilization and unloading periods were important factors for ankle range of motion restoration. In addition, balance, hip strength and ankle flexibility were correlated with the AOFAS Ankle–Hindfoot and OMAS scores. Thus, balance, hip strength and ankle ROM seem to be reliable indexes for assessing the functional status of people with ankle fractures after surgery, and, thus, they could therefore be incorporated into assessment and treatment sessions.

In general, these results provide important information for fully understanding the relationship between these physical abilities and the patients’ perceived functional status to have a broader view of an individual’s condition after ankle surgery.

## Figures and Tables

**Table 1:** Baseline characteristics of patients with bimalleolar ankle fractures at 6 months after surgery and control group.

	People with Bimalleolar Ankle Fractures (N = 21)	Healthy Control (N = 10)	*p*-Value
	Mean ± SD	Mean ± SD	
Age	44.1 ± 11.1	39.4 ± 8.8	>0.05
Gender (% female)	47.6	40	>0.05
Height (cm)	168.8 ± 9.2	171.8 ± 6.9	>0.05
Weight (kg)	77.9 ± 17.0	65.4 ± 8.2	>0.05
Injury mechanism (% low trauma)	85.5		
Infrasyndesmal fracture 44A (%)	4.8		
Transyndesmal fracture 44B (%)	66.6		
Suprasyndesmal fracture 44C (%)	28.6		
Most common ORIF employed (%)	33.3		
Immobilization time (weeks)	3.4 ± 1.2		
Unloading period (weeks)	6.1 ± 1.3		
Rehabilitation time (months)	3.2 ± 2.5		

SD: standard deviation; ORIF: open reduction and internal fixation; ORIF employed (% fibula plate with an interfragmentary screw).

**Table 2:** AOFAS Ankle–Hindfoot and OMAS scores at 6- and 12-month assessments after surgery.

People with Bimalleolar Ankle Fractures (N = 21)			Mean ± SD	Mean Diference ± SD (Lcl–Ucl)		*p*-Value
**AOFAS** **Ankle–Hindfoot**	Total score	6 months	73.7 ± 11.5	10.8 ± 9.3 (6.5; 14.9)		<0.001
		12 months	84.4 ± 12.4			
	Domain pain	6 months	28.1 ± 6.8	2.0 ± 6.0 (0.7; 4.7)		0.09
		12 months	30.5 ± 8.0			
	Domain function	6 months	36.1 ± 6.5	8.4 ± 4.7 (6.24; 10.5)		<0.001
		12 months	44.4 ± 5.0			
	Domain alignment	6 and 12 months	9.5 ± 1.5			
**OMAS**	Total score	6 months	57.0 ± 22.1	22.6 ± 13.0 (16.7; 28.5)		<0.001
		12 months	80.0 ± 25.0			

AOFAS: American Orthopaedic Foot and Ankle Society Ankle–Hindfoot score; OMAS: Olerud Molander Ankle Score; SD: standard deviation; Lcl: lower confidence limit set at 95%; Ucl: upper confidence limit set at 95%.

**Table 3:** Static balance at 6- and 12-month assessments after surgery.

		People with Bimalleolar Ankle Fractures		Healthy Controls		Asymmetry between Limbs		
						People with Bimalleolar Ankle Fractures		Healthy Controls
		6 Months	12 Months			6 Months	12 Months	
**Single leg with open eyes**	**DIS_COP_ (mm)**							
	Ol	733.8 ± 326.5 ^B^	680.6 ± 234.4	Dl	571.7 ± 274.0	103.8 (0.9; 216.5)	82.1 (−25.4; 151.7)	11.0 (−127.4; 150.0)
	Non-Ol	630.7 ± 236.5 ^C^	762.7 ± 287.4	Non-Dl	560.7 ± 250.0			
	**MV_COP_ (mm/s)**							
	Ol	24.4 ± 10.7	23.0 ± 7.8	Dl	18.5 ± 7.0	3.1 (−0.4; 6.7)	2.4 (−0.8; 5.1)	0.1 (−0.1; 0.3)
	Non-Ol	21.0 ± 7.4	25.4 ± 9.5	Non-Dl	17.6 ± 6.7			
	**LFS**							
	Ol	1.6 ± 0.7 ^A^	1.6 ± 0.8	Dl	2.3 ± 1.3	−0.2 (−0.6; 0.4)	−0.1 (−0.6; 0.4)	0.3 (−1.0; 0.5)
	Non-Ol	1.8 ± 0.8	1.5 ± 0.6	Non-Dl	2.0 ± 0.7			
**Tandem position with open eyes**	**DIS_COP_ (mm)**							
	Ol	799.2 ± 325.7 ^A,B^	675.8 ± 289.1	Dl	516.3 ± 253.9	228.6 (63; 386.0)	23.5 (−106.1; 153.2)	−13.3 (−190.0; 164.1)
	Non-Ol	570.6 ± 304.9	699.3 ± 310.7	Non-Dl	529.3 ± 165.9			
	**MV_COP_ (mm/s)**							
	Ol	26.6 ± 27.4 ^A,B^	22.4 ± 9.6	Dl	16.4 ± 5.0	7.5 (2.1; 12.8)	0.1 (−3.5; 5.1)	−1.4 (−5.4: 2.5)
	Non-Ol	19.0 ± 10.0	22.3 ± 10.0	Non-Dl	17.7 ± 6.3			
	**LFS**							
	Ol	1.3 ± 0.5	1.2 ± 0.6	Dl	1.5 ± 0.9	−0.02 (−0.4; 0.4)	0.04 (−0.6; 0.3)	0.04 (−0.4; 0.5)
	Non-Ol	1.3 ± 0.9	1.2 ± 0.7	Non-Dl	1.4 ± 0.6			

Ol: Operated limb; Non-Ol: Non-operated limb; Dl: dominant liimb; Non-Dl: Non-dominant lim; DIS_COP_: distance center of pressure; MV_COP_: velocity average of center of pressure; LFS: length/surface. Descriptives are presented as mean ± standard deviation. Asymmetry between limbs is presented as a mean (lower confidence limit at 95%; upper confidence limit at 95%). ^A^ Differences with respect to healthy control group. ^B^ Differences between operated and non-operated limbs. ^C^ Differences between 6- and 12-month assessments.

**Table 4:** Y-Balance test at 6- and 12-month assessments after surgery in participants with bimalleolar ankle fractures compared to healthy control.

	People with Bimalleolar Ankle Fractures *		Healthy Controls		Asymmetry between Limbs		
					People with Bimalleolar Ankle Fractures *		Healthy Controls
	6 Months	12 Months			6 Months	12 Months	
**Normalized reach distance (%) obtained from the anterior direction**							
Ol	56.4 ± 13.3 ^A,B,C^	61.3 ± 10.7 ^A,B^	Dl	68.0 ± 5.4	−9.4 (6.0; 12.7) ^A^	−5.6 (2.4; 8.7) ^A^	1.3 (−5.6; 3.0)
Non-Ol	65.8 ± 12.3	66.9 ± 8.4	Non-Dl	66.7 ± 8.6			
**Normalized reach distance (%) obtained from the posteromedial direction**							
Ol	102.2 ± 14.4	104.9 ± 11.7 ^B^	Dl	110.6 ± 13.3	−2.9 (−0.4; 6.1)	−2.5 (0.4; 6.1)	−2.2 (−2.1; 6.6)
Non-Ol	105.1 ± 13.5	107.3 ± 12.1	Non-Dl	112.0 ± 14.1			
**Normalized reach distance (%) obtained from the posterolateral direction**							
Ol	99.4 ± 14.8	102.2 ± 13.8	Dl	111.2 ± 15.5	−2.9 (−1.7; 7.6)	−2.5 (−2.3; 7.3)	2.1 (−6.8; 2.5)
Non-Ol	102.3 ± 15.7	104.7 ± 11.7	Non-Dl	109.1 ± 13.5			
**Composite score normalized reach distance (%)**							
Ol	86.6 ± 12.8 ^A,B^	90.1 ± 11.0 ^B^	Dl	96.6 ± 11.0	−4.7 (2.2; 7.3) ^A^	−3.3 (0.8; 5.8)	0.4 (−3.3; 2.5)
Non-Ol	91.3 ± 12.9	93.4 ± 10.0	Non-Dl	96.2 ± 11.5			

Ol: Operated limb; Non-Ol: Non-operated limb; Dl: dominant liimb; Non-Dl: Non-dominant lim; Descriptives are presented as mean ± standard deviation. Asymmetry between limbs is presented as the mean (lower confidence limit at 95%; upper confidence limit at 95%). ^A^ Differences with respect to healthy control group. ^B^ Differences between operated and non-operated limb. ^C^ Differences between 6- and 12-month assessments. * *n* = 20 and *n* = 21 participants in the ankle fracture group that performed this test in the posteromedial direction at 6 and 12 months after surgery, respectively. *n* = 19 participants in the ankle fracture group that performed this test in the posterolateral direction at 6 and 12 months after surgery.

**Table 5:** Relative weight analysis on the YBT and the AOFAS Ankle–Hindfoot and OMAS questionnaires.

Explained Variance (%, Adjusted R^2^)								
12 Months	Total Variance Explained		Age	Ankle DF ROM	Hip ABD	Hip ADD	Immobilization Time	Unloading Period
				6 Months	6 Months	6 Months		
**YBT_A_**	63%	**Rel. weight** **CI**		20 (2.4; 44.0)	17 (2.8; 25.1)	26 (7.0; 56.0)		
**YBT_PM_**	64%	**Rel. weight** **CI**	40 (16.0; 58.0)		24 (7.6; 4.3)			
**YBT_CS_**	60%	**Rel. weight** **CI**	40 (10.0; 63.1)		20 (1.9; 40.7)			
**AOFAS**	44%	**Rel. weight** **CI**			25 (5.4; 44.4)	19 (5.3; 29.0)		
**Domain function**	47%	**Rel. weight** **CI**		16 (2.6; 32.8)	15 (3.5; 28.4)	16 (5.1; 27.7)		
**OMAS**	35%	**Rel. weight** **CI**			18 (1.4; 40.1)	17 (2.7; 28.1)		
**ADF_ROM_**	55%	**Rel. weight** **CI**					23 (6.9; 42.7)	32 (13.1; 47.0)

Rel. weight: relative weight; CI: confidence interval at 95%; YBT_A_: Y-Balance test anterior direction; YBT_PM_: Y-Balance test posteromedial direction; YBT_CS_: Y-Balance test composite score; DF: dorsal flexion; ROM: range of movement; ABD: hip abductor strength, ADD: hip adductor strength; AOFAS: American Orthopaedic Foot and Ankle Society hindfoot score; OMAS: Olerud Molander Ankle Score.

## Data Availability

Not applicable.

## Supplementary Materials

The following supporting information can be downloaded at: https://www.mdpi.com/article/10.3390/jcm11092539/s1, Table S1: Ankle dorsal flexion ROM, hip strength, calf and bimalleolar circumferences at 6- and 12-months assessments after surgery; Table S2: Correlations among the parameters evaluated at 6 months and 12 months assessments after surgery.
