# Supplementary material for: Measuring Recovery and Understanding Long-Term Deficits in Balance, Ankle Mobility and Hip Strength in People after an Open Reduction and Internal Fixation of Bimalleolar Fracture and Their Impact on Functionality: A 12-Month Longitudinal Study
Source: J Clin Med. 2022 Apr 30;11(9):2539. doi: 10.3390/jcm11092539 (PMC9101534; doi:10.3390/jcm11092539)
Supplement: Supplementary file 1 [file jcm-11-02539-s001.zip › table S1. Suplementary information.pdf]

Table S1. *Ankle dorsal flexion ROM, hip strength, calf and bimalleolar circumferences at 6- and 12-months assessments after surgery*

	PEOPLE WITH BIMALLEOLAR ANKLE FRACTURES		HEALTHY CONTROLS		ASYMMETRY BETWEEN LIMBS		
					PEOPLE WITH BIMALLEOLAR ANKLE FRACTURES		HEALTHY CONTROLS
	**6-months**	**12-months**			**6-months**	**12-months**	
Ankle dorsal flexion ROM (°)							
**Operated Limb**	22.9 ± 7.6[A,B,C]	29.7 ± 9.2[A,B]	**Dominant Limb**	40.7 ± 4.9	-12.4 (9.9; 14.8)[A]	-7.4 (4.6; 10.3)[A]	1.3 (-3.6; 0.9)
**Non-operated Limb**	35.3 ± 5.4	37.1 ± 6.0	**Non-dominant Limb**	39.4 ± 7.0			
Hip ABD strength normalized to body mass (%)							
**Operated Limb**	25.3 ± 7.3[A,B,C]	30.2 ± 7.7	**Dominant Limb**	35.0 ± 8.9	-4.0 (1.3; 6.7)[A]	-1.9 (-0.2; 4.2)	0.9 (-3.0; 1.2)
**Non-operated Limb**	29.4 ± 8.9[C]	32.1 ± 7.3	**Non-dominant Limb**	34.0 ± 7.9			
Hip ADD strength normalized to body mass (%)							
**Operated Limb**	26.2 ± 9.3[C]	28.8 ± 8.5[B]	**Dominant Limb**	33.2 ± 9.5	0.1 (-1.6; 1.3)	2.1 (-4.1; -0.2)	2.1 (-6.8; 2.5)
**Non-operated Limb**	26.1 ± 8.7	26.6 ± 7.2	**Non-dominant Limb**	31.3 ± 8.9			
Calf circumference (cm)							
**Operated Limb**	34.3 ± 4.1[B]	34.5 ± 4.5[B]	**Dominant Limb**	34.2 ± 2.3	1.3 (0.6; 2.1)[A]	1.0 (0.2; 1.8)[A]	0.4 (-1.3; 0.6)
**Non-operated Limb**	35.6 ± 4.5	35.5 ± 4.3	**Non-dominant Limb**	33.9 ± 2.7			
Bimalleolar circumference (cm)							
**Operated Limb**	25.0 ± 2.1[A,B]	25.7 ± 2.0[A,B]	**Dominant Limb**	22.0 ± 1.7	-1.0 (-1.2; -0.8)[A]	-1.1 (-1.4; -0.8)[A]	-0.3 (-0.7; 0.2)
**Non-operated Limb**	24.0 ± 2.1	24.6 ± 1.7	**Non-dominant Limb**	21.8 ± 1.4			

ROM: range of movement; ABD: hip abductor muscle strength; ADD: hip adductor muscle strength.

Descriptives are presented as mean ± standard deviation. Asymmetry between limbs are presented as mean (lower confidence limit at 95%; upper confidence limit at 95%).

[A]Differences with respect to healthy control group; B: [B]Differences between operated and non-operated limb; [C]Differences between 6- and 12-month assessments.
