# Supplementary material for: Measuring Recovery and Understanding Long-Term Deficits in Balance, Ankle Mobility and Hip Strength in People after an Open Reduction and Internal Fixation of Bimalleolar Fracture and Their Impact on Functionality: A 12-Month Longitudinal Study
Source: J Clin Med. 2022 Apr 30;11(9):2539. doi: 10.3390/jcm11092539 (PMC9101534; doi:10.3390/jcm11092539)
Supplement: Supplementary file 1 [file jcm-11-02539-s001.zip › Table S2. Suplementary information.pdf]

Table S2. Correlations among the parameters evaluated at 6 months (from left to right, color: light grey) and 12 months (from up to down, color: white) assessments after surgery

	YBT$_{A}$	YBT$_{PM}$	YBT$_{CS}$	SL-OL$_{EO}$ DIS$_{COP}$	TD-OL$_{EO}$ DIS$_{COP}$	TD-OL$_{EO}$ MV$_{COP}$	Age	Immob. time	Unloading period	Reh. time	CP	BMP	ADF$_{ROM}$	H$_{ABD}$	H$_{ADD}$	AOFAS$_{AH}$ Function	AOFAS$_{AH}$ Total score	OMAS
YBT$_{A}$		0.705**	0.862**	-0.124	0.174	0.150	-0.500*	-0.275	-0.352	-0.516*	-0.194	-0.122	0.535*	0.737**	0.773**	0.674**	0.637**	0.547*
YBT$_{PM}$	0.648**		0.904**	0.084	-0.126	-0.052	-0.575**	-0.347	-0.178	-0.372	-0.008	-0.104	0.341	0.394	0.427	0.345	0.290	0.090
YBT$_{CS}$	0.815**	0.912		-0.133	-0.082	-0.010	-0.597**	-0.337	-0.260	-0.540*	-0.177	0.079	0.664**	0.664**	0.409	0.378	0.325	-0.055
SL-OL$_{EO}$ DIS$_{COP}$	-0.129	0.093	-0.044		0.709	0.482*	0.242	0.368	0.225	0.128	-0.312	0.485*	-0.162	-0.070	-0.036	-0.266	-0.197	-0.121
TD-OL$_{EO}$ DIS$_{COP}$	-0.140	-0.214	-0.035	0.226		0.749**	0.358	0.476*	0.446*	0.150	-0.151	0.442	-0.432*	-0.055	-0.049	-0.309	-0.178	-0.130
TD-OL$_{EO}$ MV$_{COP}$	-0.304	-0.214	-0.261	-0.037	0.271		0.272	-0.094	-0.263	0.154	-0.419	0.354	-0.541*	-0.092	-0.218	-0.031	-0.096	-0.106
Age	-0.501*	-0.717**	-0.708**	0.254	0.301	0.301		0.322	0.218	0.381	-0.180	-0.092	-0.295	-0.374	-0.494*	-0.402	-0.467*	-0.396
Immob0. time	-0.285	-0.374	-0.259	0.354	0.327	0.327	0.322		-0.009	0.020	-0.032	0.413	-0.438*	-0.067	-0.226	-0.162	-0.014	0.016
Unloading period	-0.380	-0.416	-0.412	0.072	0.131	0.131	0.218	0.020		0.135	0.229	-0.158	-0.467*	-0.322	-0.381	-0.302	-0.198	-0.261
Reh0. time	-0.448*	-0.257	-0.518*	0.053	0.176	0.176	0.381	-0.009	0.135		-0.286	-0.126	-0.598**	-0.235	-0.267	-0.815**	-0.783**	-0.553*
CP	0.217	0.180	0.402	0.004	-0.136	-0.136	0.036	-0.167	-0.010	0.229		-0.012	0.000	-0.566*	-0.384	0.038	0.247	-0.084
BMP	0.044	0.085	0.262	-0.081	-0.007	-0.007	0.176	0.031	-0.141	-0.019	0.597**		0.016	0.028	-0.043	-0.116	-0.106	-0.193
ADF$_{ROM}$	0.685**	0.568**	0.320	-0.139	-0.268	-0.268	-0.414	-0.480*	-0.561**	-0.349	0.450*	-0.044		0.434*	0.326	0.647**	0.479*	0.451*
H$_{ABD}$	0.613**	0.638**	0.594**	0.193	-0.329	-0.330	-0.499*	-0.193	-0.360	-0.216	0.253	-0.008	0.510*		0.812**	0.465*	0.403	0.592**
H$_{ADD}$	0.718**	0.722**	0.514*	0.227	-0.305	-0.305	-0.454*	-0.323	-0.312	-0.384	0.178	0.127	0.659**	0.841**		0.259	0.472*	0.582**
AOFAS$_{AH}$ Function	0.496*	0.302	0.490*	0.379	-0.131	-0.131	-0.236	-0.231	-0.364	-0.415	-0.054	-0.078	0.751**	0.557**	0.743**		0.896**	0.786**
AOFAS$_{AH}$ total score	0.454*	0.235	0.357	0.430	-0.018	-0.019	-0.153	-0.060	-0.194	-0.372	-0.112	-0.087	0.632**	0.483*	0.716**	0.940**		0.853**
OMAS	0.477*	0.247	0.092	0.387	-0.026	-0.027	-0.250	0.033	-0.166	-0.346	-0.216	-0.103	0.564**	0.465*	0.642*	0.819**	0.892**	

*$p<0.005$. ** $p<0.001$. YBT$_{A}$: Y-balance Test anterior direction; YBT$_{PM}$: Y-balance Test posteromedial direction; YBT$_{CS}$: Y-balance Test Composite score; SL-OL$_{EO}$: single-leg stance with the operated limb with eyes open; TD-OL$_{EO}$: tandem position operated limb with eyes open; DIS$_{COP}$: center of pressure length path; MV$_{COP}$: average speed of center of pressure; Immob.time: immobilization time; Reh.time: rehabilitation time; CP: calf circumference; BMP: bimalleolar circumference; ADF$_{ROM}$: range of ankle dorsal flexion movement; H$_{ABD}$: hip abduction strength; H$_{ADD}$: hip adduction strength; AOFAS: American Orthopedic Foot and

Ankle Society Ankle-Hindfoot Score; OMAS: Olerud Molander Ankle Score. Correlations were only performed on the operated limb and the balance tasks that showed significant differences between the operated and non-operated limb at 6- and 12- months assessment after surgery
